# Fracture of the Greater Process of the Calcaneum

**Published:** 1903-01-10

**Authors:** 


### FRACTURE OF THE GREATER PROCESS OF THE CALCANEUM.

There are few points on which radiography has thrown more light than in regard to the nature of those obscure fractures or "crushes" of the bones of the tarsus which are so often regarded as mere sprains or contusions. In an article in the *Practitioner* for this month, by Carless and Mayou, it is pointed out that radiography has demonstrated that there is a fairly common fracture of the os calcis which results from falls on the feet. The essential element in the violence that produces this fracture is a fall from a height on the heels, the patient alighting either in the standing or squatting position. Falls down the shafts of lifts, jumping from windows, or dropping from telegraph poles, etc., may be mentioned as special types of accidents leading to this lesion. Of course other fractures may result from similar violence, for slight differences in the strength and elasticity of the bones, or in the exact direction of the force may result in very different injuries. Under ordinary circumstances when an individual is standing at rest the weight of the trunk transmitted through the astragalus, which forms the keystone of the arch of the foot, and when a person falls plump on the feet from a height it is probable that the force is mainly spent upon the astragalus, fracture and even comminution of which bone is likely to occur. Should the violence, however, be applied not quite vertically, then a greater portion will be directed either to the anterior or posterior segment of the arch, and in the first of these cases the form of fracture referred to by the authors is likely to take place. "The head of the astragalus," they say, "rests inferiorly upon the sustentaculum tali, and also to a lesser extent upon the greater process of the calcaneum. Violence applied vertically would force these two surfaces together, but in addition to this the anterior edge of the posterior facet on the under surface of the astragalus would be driven downwards into the outer end of the groove into which is inserted the interosseous ligament," and the result may be to break the greater process of the calcaneum across, the anterior fragment sometimes being comminuted, while sometimes impaction occurs. Flattening of the arch of the foot will be the necessary consequence of this lesion, and according to the extent and direction of the violence, other injuries, such as crushing and splitting of the astragalus may be added to it. The immediate clinical signs of these cases are not very characteristic. The ecchymosis is usually situated a little above the fracture, since the tissues immediately below the malleoli swell more readily than the plantar structures. The heel is generally somewhat drawn up. The loss of the arch of the foot is not very evident, owing to the swelling, but the foot looks longer than usual, and its vertical dimensions may be diminished. It is all important in these cases to take radiographs not only from the side but also from the front. The treatment of this injury is somewhat unsatisfactory. When the ecchymosis is considerable a bandage and evaporating lotion may be applied until the swelling has subsided, when, if the displacement is considerable and the arch of the foot much depressed, it is advisable to force the foot into a position of inversion under an anæsthetic, either by hand or by Thomas's wrench, and put it up in plaster of Paris. If the deformity should be great, massage may be used, and later the employment of a slight instep pad may possibly enable the patient to walk without much pain. In old-standing cases of pain or deformity from this cause it may be possible to remove a wedge of bone irrespective of joints, or to excise the whole or part of the astragalus.

